# Shared Governance as an Interprofessional Decision-Making System in Hospitals: Implementation and Evaluation Challenges

**DOI:** 10.2147/JHL.S646651

**Published:** 2026-10-30

**Authors:** Christoph Golz, Kai-Uwe Schmitt, Mirjam Körner

**Affiliations:** 1 School of Health Professions, Bern University of Applied Sciences, Bern, Switzerland

**Keywords:** shared governance, professional governance, interprofessional governance, hospital leadership, implementation, measurement, decision-making

## Abstract

Shared governance has been recognized for more than three decades as an organizational framework for strengthening nurses’ participation in decision-making, professional autonomy, and accountability. In recent years, the concept has regained attention as healthcare organizations face workforce shortages, increasing care complexity, and growing demands for interprofessional collaboration. This Expert Opinion examines the development, implementation, and evaluation of shared governance from nursing-centered models toward broader professional and interprofessional governance systems. Building on existing conceptual and measurement work, it proposes a conceptual framework for understanding shared governance as an explicit interprofessional decision-making system that links governance context, structures, operations, decision pathways, workforce, patient, and organizational outcomes. Implementation is complicated by conceptual overlap with frameworks such as nursing excellence recognition programs (eg, Magnet®), distributed leadership, clinical governance, quality improvement structures, and interprofessional collaboration. These overlaps may blur responsibilities, duplicate structures, and complicate the attribution of outcomes to shared governance. Measurement approaches have advanced but remain fragmented. Instruments such as the Index of Professional (Nursing) Governance and the Verran Professional Governance Scale assess perceptions of governance culture, while the Council Health Survey provides a council-level measure of council functioning. However, current measurement approaches do not yet sufficiently capture interprofessional governance as an operational system, including how authority is distributed across professional groups, how decisions move through organizational structures, and how governance contributes to workforce, patient, and organizational outcomes. Based on this framework, we propose a research and practice agenda that emphasizes conceptual clarification, interprofessional alignment with existing organizational frameworks, measurement innovation, and rigorous implementation and evaluation designs. Future work should combine measures of governance culture, council functioning, interprofessional representation, decision pathways, structural characteristics, and outcomes. Such an approach can help shared governance move from symbolic or fragmented implementation toward a measurable and sustainable mechanism for strengthening professional engagement, workforce retention, interprofessional collaboration, and the quality and safety of patient care.

## Introduction

Healthcare systems worldwide are facing increasing workforce shortages, rising care complexity, and growing demands for high-quality, patient-centered services.^1^ These pressures are not only questions of staffing levels, but also of how hospitals organize authority, coordination, and decision-making across professional and organizational boundaries. Hospitals must coordinate multidisciplinary care, maintain patient safety, and make timely decisions across complex care pathways. Staffing gaps endanger the quality, safety, and continuity of care and intensify the strain on existing staff.^2–4^ These workforce challenges are compounded by chronic occupational stressors, including high workloads, emotional burden, time pressure, and limited opportunities for professional development.^5–8^ Such factors are strongly associated with burnout, job dissatisfaction, and premature attrition from the nursing profession, further exacerbating workforce instability.^7–10^

In this context, shared governance models that promote professional autonomy, shared decision-making, and participatory leadership have gained renewed attention as levers for workforce retention and quality improvement.^11–13^ Shared governance is a structural framework that empowers staff to actively participate in decision-making regarding clinical practice, resource allocation, and organizational strategy. Grounded in the principles of partnership, equity, accountability, and ownership, shared governance aims to decentralize authority, promote transparent communication, and foster collaboration between frontline staff and management.^14–17^ Shared governance has since been recognized as a key component of nursing excellence recognition programs (eg, Magnet®) and other high-performing healthcare systems.^11^,^16^,^18^

In recent years, the organizational challenge shared governance addresses has evolved. Hospitals increasingly depend on integrated care pathways, multidisciplinary teams, distributed authority, and cross-professional decision-making to address complex patient needs and safety requirements.^19^,^20^ As hospitals move toward more integrated, interdisciplinary models of care delivery, the role of shared governance is expanding beyond its nursing roots toward broader interprofessional governance structures.^21^,^22^

Despite this renewed attention, substantial conceptual and practical challenges remain. Much of the existing evidence still focuses on descriptive or correlational assessments of perceptions of governance rather than on causal evaluations or implementation research. Furthermore, the concept of shared governance increasingly overlaps with other leadership and quality frameworks, such as nursing excellence recognition programs, distributed leadership, clinical governance, quality improvement teams, and interprofessional collaboration frameworks. This overlap creates ambiguity regarding responsibilities, decision rights, and evaluation.^23–25^ At the same time, the expansion of shared governance beyond nursing raises additional questions about professional representation, authority distribution, and the integration of interprofessional decision-making structures within existing hospital governance systems.

Existing work has advanced understanding of shared governance, professional governance, council functioning, and interprofessional collaboration.^25–30^ However, these bodies of literature are often discussed separately and provide limited guidance on how shared governance can be operationalized as an interprofessional decision-making system in hospitals. This expert opinion is based on selected conceptual and empirical literature. We considered key sources because they define central concepts, describe implementation experiences, report measurement approaches, or illustrate challenges in linking governance structures to workforce, patient, and organizational outcomes. The article contributes to the current debate by linking these dimensions into a conceptual perspective on shared governance as an interprofessional decision-making system.

## Development of Shared Governance in Hospitals

The concept of shared governance emerged in the late 1980s and early 1990s as a response to growing calls for enhanced professional autonomy and participatory decision-making in nursing.^14^,^31^ Early shared governance models were primarily designed to strengthen nurses’ professional roles within hospital hierarchies by decentralizing authority, empowering frontline staff, and formalizing staff participation in clinical and organizational decision-making.^14–16^,^25^,^32^ Rooted in democratic leadership principles, these models sought to create organizational structures through which nurses could influence decisions on practice standards, policies, quality improvement, resource allocation, and professional development.^16^,^25^

The original models were therefore primarily nursing-centered. They often established nursing practice councils, resource councils, personnel councils, or similar structures at unit, departmental, and organizational levels. Decision-making authority was intentionally shifted away from traditional management hierarchies toward structures in which nurses could share control over clinical practice and related organizational domains.^14^,^31^ These structures were expected to cultivate accountability, strengthen professional identity, and improve alignment between frontline practice and organizational strategy.^15^,^25^,^32^

Empirical work in this field has often focused on descriptive evaluations of governance structures and staff perceptions of empowerment, autonomy, and participation. The Index of Professional Nursing Governance (IPNG) was developed to measure the extent to which nurses perceive their organization as traditionally governed, shared governed, or self-governed across domains, including personnel, information, resources, participation, practice, and goals.^33^ Studies using the IPNG and related approaches have linked stronger shared governance perceptions to higher job satisfaction, organizational commitment, empowerment, and perceived autonomy.^14^,^33^ However, evidence on patient outcomes and causal pathways remains limited. The few available studies nevertheless suggest that shared governance may be relevant for quality and safety. Nurse engagement in shared governance has been associated with more favorable nurse-reported ratings of quality of care and patient safety, and studies of professional nursing governance have linked stronger governance to selected nurse-sensitive and patient satisfaction outcomes. These findings support the potential relevance of shared governance for quality and safety but remain largely observational and nursing-focused.^34^,^35^

A broader understanding of governance was introduced through the concept of professional governance. Rather than replacing shared governance, professional governance can be understood as a broader conceptual framework that encompasses the structures and processes by which professionals direct, control, and regulate their goal-oriented work within organizations.^18^,^36^ In this context, the Index of Professional Governance (IPG) extends measurement beyond nursing, allowing governance to be assessed across professional groups.^26^ This distinction is important because contemporary hospitals rely on interdependent clinical and non-clinical professions whose decisions increasingly affect shared care pathways, patient safety, resource use, and organizational performance.

The renewed relevance of professional and interprofessional governance reflects the growing complexity of healthcare delivery. As hospitals move toward integrated and team-based care, governance structures are increasingly expected to transcend disciplinary boundaries and include physicians, allied health professionals, and other clinical and managerial stakeholders.^21^,^22^,^27^,^37^,^38^ Interprofessional councils or shared decision-making bodies may address issues such as care pathway design, resource prioritization, digital transformation, quality improvement, and workforce development across professional boundaries.

Empirical work provides initial examples of this interprofessional turn. For instance, longitudinal work on interprofessional shared governance has linked implementation efforts to staff outcomes such as workplace empowerment and engagement over time. However, such studies also illustrate the current limitations of the evidence base, as interprofessional governance structures are often evaluated using outcomes and samples that remain strongly shaped by nursing participation.^39^

This development also creates new implementation and evaluation challenges. Although the IPG provides a measurement approach for professional governance beyond nursing, much of the empirical literature still relies on nursing-focused instruments, nursing-only samples, or descriptive accounts of governance structures. Moreover, culture and perception measures alone do not show whether councils have clear mandates, whether decision rights are explicit, how decisions are escalated, or whether council decisions lead to measurable changes in practice and outcomes. Thus, the challenge is not simply the absence of interprofessional measurement tools, but the need to evaluate shared governance as an operational decision-making system embedded in complex hospital structures.

This development from nursing-centered shared governance toward professional and interprofessional governance, therefore, requires more than terminological refinement. It requires conceptual clarity, explicit alignment with existing organizational frameworks, and evaluation approaches that capture not only perceptions of governance culture, but also the structure, functioning, and consequences of governance processes in practice.

## Overlapping Frameworks: A Hidden Barrier

While shared governance has evolved conceptually and structurally over the past decades, its implementation and evaluation in contemporary hospitals are increasingly complicated by overlap with other organizational and leadership frameworks. Hospitals often operate within multiple governance, quality, and leadership structures. When shared governance is introduced or expanded, particularly in interprofessional contexts, it may intersect with pre-existing frameworks, resulting in blurred boundaries, duplicated committees, and ambiguous decision-making rights.

Several frameworks are particularly relevant in this regard: nursing excellence recognition programs; participative and distributed leadership frameworks; quality improvement and patient safety structures; clinical governance; interprofessional collaboration frameworks; and traditional hierarchical decision-making bodies. Table 1 summarizes these overlaps, common points of confusion, and their implications for implementation and evaluation.

**Table 1:** Overlaps Between Shared Governance and Related Organizational Frameworks in Hospitals

Framework	Overlap with Shared Governance	Typical Confusion Points	Consequences for Implementation	Consequences for Evaluation
Recognition Program Model (ANCC)	Shared governance is embedded within recognition program components (eg, Structural Empowerment, Transformational Leadership).	Hospitals implement recognition programs as bundled programs, assuming that governance is implicitly addressed.	Councils may be created superficially to meet recognition program requirements, without clear roles or decision rights.	The effects of shared governance are difficult to isolate from those of broader nursing excellence recognition interventions.
Participative/ Distributed Leadership	Emphasizes staff engagement and shared decision-making similar to governance.	Terms used interchangeably; unclear delineation between leadership roles and council authority.	Parallel structures may emerge, or roles may merge without clarity.	Measurement tools capture overlapping constructs (eg, empowerment), complicating attribution.
Quality Improvement (QI) and Patient Safety Structures	QI teams address similar operational issues as governance councils.	Ambiguity over which structure handles problem-solving and implementation.	Councils risk marginalization if QI teams dominate operational decisions.	Improvements may be attributed to QI initiatives rather than governance structures.
Clinical Governance	Focuses on accountability, quality, and risk management; overlaps with governance domains.	“Governance” is used generically, without distinguishing shared vs clinical governance.	Ambiguous reporting lines and committee redundancies.	Conceptual confusion undermines clarity in evaluation.
Interprofessional Collaboration Frameworks	Promote shared decision-making and team responsibility across professions.	Governance councils broadened without adapting charters; overlap with IPC teams.	Misalignment between nursing-led councils and interprofessional forums.	Collaboration metrics are often used as proxies for governance metrics.
Organizational/ Hierarchical Decision Structures	Governance is meant to bridge the frontline and management decision levels.	Overlapping committees (executive boards, strategy groups) may make similar decisions.	Councils lack decision authority and become advisory only.	Governance maturity is difficult to assess if formal and actual decision flows diverge.

From a research perspective, these overlaps make it challenging to attribute observed outcomes (eg, improved staff satisfaction, reduced turnover, improved quality indicators) to shared governance structures per se. Many studies simply report the presence of nursing excellence recognition program designation or shared governance councils without specifying how these interact with other frameworks.^18^,^23^,^24^ This lack of conceptual clarity represents a major barrier to building robust evidence on the effectiveness of shared governance.

## Barriers and Facilitators of Implementation

Despite the long-standing presence of shared governance in the nursing literature, its implementation in hospital practice remains uneven, and many initiatives fail to reach their intended impact. Evidence from the scoping review and implementation frameworks such as CFIR and i-PARIHS suggests that barriers and facilitators occur across multiple domains, including intervention design, organizational context, individual characteristics, and implementation processes.^23^,^24^,^28^,^37^,^38^ This becomes particularly relevant when shared governance is expanded beyond nursing, as interprofessional governance requires role clarity, explicit allocation of authority, effective communication, and organizational support.^29^

### Barriers

#### Lack of Conceptual Clarity and Framework Overlap

As discussed in the previous section, overlapping frameworks such as nursing excellence recognition programs, clinical governance, and QI structures often result in unclear responsibilities and decision-making pathways. When governance councils are introduced without explicitly defining their relationship to existing structures, decision-making authority remains ambiguous. Councils may become symbolic rather than functional, eroding staff trust and engagement.^30^,^40^

#### Insufficient Leadership Continuity and Support

Shared governance requires consistent leadership endorsement, dedicated resources, and protected time for staff to participate in councils. Frequent changes in executive or nursing leadership can destabilize governance structures, leading to loss of momentum and unclear priorities. Lack of visible support from senior management undermines the perceived legitimacy of governance councils, discouraging active participation by staff.^18^,^41–43^

#### Resource and Time Constraints

Clinical workload pressures and staffing shortages often limit the capacity of nurses and other professionals to engage in governance activities. Councils that meet infrequently or rely on ad hoc participation struggle to maintain continuity and implement decisions effectively. Without protected time, governance participation is often perceived as an “extra” rather than a core professional responsibility.^43^

#### Role Ambiguity and Limited Decision Rights

In many hospitals, governance structures are introduced without a clear definition of decision-making authority. Councils may provide non-binding recommendations, leading to frustration among participants when decisions are overturned by management hierarchies.^30^ This lack of authority undermines accountability and can result in governance fatigue.

#### Measurement and Feedback Gaps

Implementation efforts often lack clear evaluation plans or feedback loops. Councils may generate recommendations, but the impact of these recommendations on clinical practice, workforce outcomes, or organizational performance remains undocumented. Without visible feedback, staff lose trust in the governance process and question its relevance.^30^,^40^

### Facilitators

#### Executive Sponsorship and Visible Leadership Commitment

Strong support from nursing and executive leadership is consistently identified as a key enabler of effective governance.^18^,^30^,^43^ This includes allocating resources, integrating council recommendations into strategic decision-making, and visibly endorsing council outputs. When leaders actively participate in or respond to governance activities, councils gain legitimacy and influence.

#### Structural Clarity and Alignment

Successful implementations explicitly define the mandate, authority, and scope of governance councils and align them with existing organizational structures. Clear charters, bylaws, and decision pathways prevent overlap with QI or hierarchical bodies and make responsibilities transparent.^18^,^30^,^40^

#### Capacity Building and Role Development

Training staff to lead councils, chair meetings, analyze data, and navigate interprofessional dynamics strengthens the implementation of governance.^18^,^43^,^44^ Empowering frontline health professionals to take on leadership roles within governance structures supports both individual development and organizational capacity by fostering ownership, confidence, reflective practice, and leadership competence.

#### Feedback Mechanisms and Outcome Linkage

Embedding governance within existing reporting and quality structures, so that council decisions lead to visible changes and measurable outcomes, is essential for sustaining engagement.^18^,^40^ Transparent feedback loops demonstrate the value of participation, strengthen the perceived legitimacy of governance councils, and help councils refine their work over time.

## Measurement Challenges

Measurement challenges arise because existing approaches capture important but different dimensions of shared and professional governance, including governance culture, council functioning, and selected outcomes. However, these dimensions are rarely integrated into a comprehensive evaluation of governance as an operational decision-making system.

### From Governance Culture to Governance Operations

Shared governance evaluation benefits from distinguishing between measuring governance culture and measuring council functioning. The IPNG and its interprofessional extension, the IPG, are designed to demonstrate the presence and degree of shared governance across an organization.^30^ They can be used at baseline and in follow-up assessments to monitor progress along a governance continuum, and they have been applied across many organizations and settings.^26^ In contrast, the work of shared governance, namely the deliberation and decisions that change practice, takes place within councils. Consequently, an organization may demonstrate an overall shared governance culture while still having councils that function suboptimally, which can lead to frustration, low productivity, and limited visible impact.^30^

To address this gap, Hess et al developed the Council Health Survey as a quantitative tool to assess council effectiveness.^30^ The Council Health Survey comprises 25 items grouped into three subscales: structure, activities, and membership.^30^ It operationalizes core features of effective councils, including clarity of charters and bylaws, leadership engagement, evidence and data use, decision-making processes, stakeholder communication, and processes for selecting, preparing, and replacing members.^30^ In practical terms, combining organization-level governance culture measures with council-level functioning measures can improve implementation fidelity assessment and can support learning loops that link governance structures to measurable change in practice and outcomes.^30^

### Lack of Structural and Outcome Measures

Although the IPNG remains a central instrument for assessing nursing governance, the broader evaluation challenge lies in moving beyond perception-based measures toward structural, process, and outcome indicators. Beyond perception surveys and selected council-level tools, few standardized approaches exist to measure structural characteristics, such as the number and types of councils, decision rights, and escalation pathways, or organizational outcomes directly attributable to governance. Many studies continue to rely on cross-sectional designs, descriptive reports, or governance perception measures, while systematic tracking of governance activities and their impact on clinical practice or organizational performance remains limited.^28^,^30^,^45^ Similarly, few studies integrate governance variables into multi-level datasets that include staff outcomes, such as retention and satisfaction, patient outcomes, such as quality indicators and safety events, and organizational metrics, such as turnover or financial performance.

This lack of standardized structural and outcome measures hinders the ability to compare governance models across organizations or to evaluate longitudinal changes. It also limits the integration of governance evaluation into broader quality and performance monitoring systems.^28^,^45^

## Interprofessional Shared Governance as a Decision-Making System

The preceding sections suggest that interprofessional shared governance in hospitals should not be understood only as a general culture of participation or as the presence of councils. We propose conceptualizing it as an interprofessional decision-making system in which context, structures, operations, and outcomes are connected. In this framework, governance context includes workforce complexity, multidisciplinary care, patient safety demands, and overlapping organizational frameworks such as nursing excellence recognition programs, clinical governance, quality improvement, distributed leadership, and interprofessional collaboration. Governance structures refer to the formal arrangements that organize participation and authority, including council architecture, decision domains, decision rights, authority distribution, interprofessional representation, leadership support, and protected resources. Governance operations describe how shared governance becomes active in practice through council functioning, deliberation, escalation pathways, communication, feedback loops, evidence use, and implementation of council decisions. Finally, evaluation should link these contextual, structural, and operational dimensions to workforce, patient, and organizational outcomes. Figure 1 summarizes this proposed framework and illustrates interprofessional shared governance as a decision-making system embedded within complex hospital structures.

**Figure 1:**
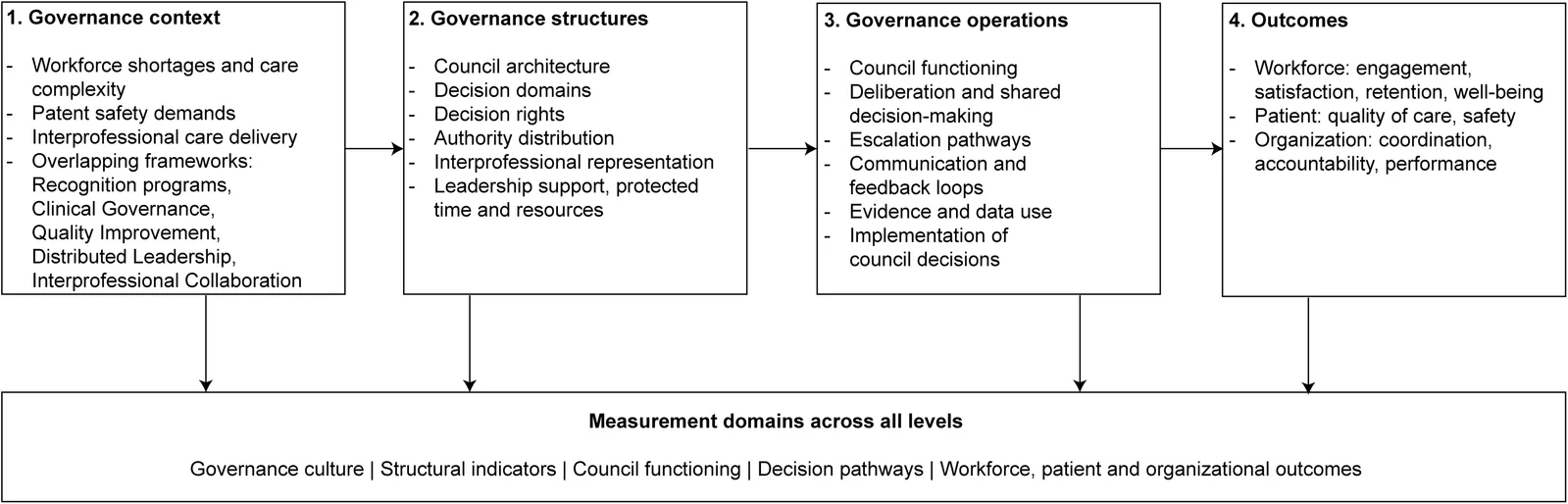
Shared governance as an interprofessional decision-making system in hospitals.

The figure illustrates shared governance as an interprofessional decision-making system shaped by governance context and overlapping organizational frameworks. The boxes represent key domains derived from the selected literature, whereas the arrows indicate the authors’ proposed conceptual linkages between these domains. Contextual conditions influence governance structures, which in turn shape governance operations and decision-making processes. These processes may contribute to workforce, patient, and organizational outcomes. The framework also highlights the need to evaluate shared governance across multiple domains, including governance culture, structural indicators, council functioning, decision pathways, and outcomes.

## Research and Practice Agenda

The renewed interest in shared governance offers a timely opportunity to advance both research and practice toward a more conceptually precise, structurally explicit, and methodologically robust approach. Building on the arguments developed in the previous sections, we propose that shared governance should be studied and implemented not only as a participatory leadership model, but as an organizational decision-making system embedded within complex hospital structures. Accordingly, future work should address three key priorities: (1) clarifying and aligning governance structures, (2) innovating measurement approaches, and (3) applying rigorous implementation and evaluation designs.

### Clarifying Conceptual Foundations

A fundamental step toward advancing shared governance is to define its conceptual boundaries and organizational position within hospitals. Organizations should specify the decision domains, authority levels, and professional groups included in governance processes, and distinguish shared governance from related frameworks such as nursing excellence recognition programs, clinical governance, distributed leadership, QI structures, and interprofessional collaboration frameworks. This clarification should be embedded in organizational policies, council charters, and communication strategies to avoid ambiguity and ensure accountability.^26^,^30^,^40^

At the same time, shared governance structures should be explicitly mapped and aligned with existing leadership, quality, and clinical governance structures. QI teams and governance councils should have complementary, not competing, responsibilities, with clear escalation pathways and shared accountability. Recognition-program structures should support, rather than overshadow, governance councils, and interprofessional collaboration frameworks should be harmonized with governance charters to ensure broad professional representation.^16^,^27^,^40^

For research and evaluation, this conceptual and structural alignment is essential. Rather than relying on broad notions of empowerment or participation, future studies should specify the mechanisms through which governance is expected to influence staff, patient, and organizational outcomes. Logic models or program theories can help clarify these mechanisms and guide implementation and evaluation.^37^,^38^,^46^

However, conceptual and structural clarification alone is unlikely to ensure successful implementation. The expansion of shared governance to interprofessional settings also requires attention to contextual conditions and potential unintended consequences. Interprofessional governance may function differently across healthcare systems, hospital types, and organizational cultures. Hierarchical cultures, limited psychological safety, insufficient resources, and weak leadership support may restrict meaningful participation and reduce councils to advisory rather than decision-making bodies.^18^,^28^,^43^ Professional power dynamics also require explicit consideration. If authority distribution and decision rights are not clearly defined, interprofessional councils may reproduce existing hierarchies, privilege dominant professional groups, or frustrate participants whose recommendations are not implemented.^29^,^30^,^40^

Expanding shared governance may also increase organizational complexity. Additional councils or committees can duplicate existing quality improvement, clinical governance, recognition-program, or managerial structures if their mandates and escalation pathways are not clearly aligned.^23^,^24^,^30^,^40^ In such cases, shared governance may create competing authority structures rather than improving decision-making. These risks underline why implementation should include clear council charters, defined decision domains, protected time and resources, feedback mechanisms, and explicit alignment with existing governance structures.^18^,^30^,^40^,^43^ From an evaluation perspective, implementation fidelity, mechanisms of action, and contextual moderators should also be assessed.^37^,^38^,^46^

### Innovating Measurement Approaches

The field, therefore, urgently needs measurement tools that reflect modern governance realities. Future research should focus on:

- Developing interprofessional governance measurement instruments, either by adapting existing nursing-focused tools (eg, IPNG, VPGS) or creating new instruments that capture the perspectives of multiple professional groups.
- Standardizing structural indicators to describe governance models in a comparable way across organizations.
- Integrating governance metrics into routine organizational data systems enables continuous monitoring and feedback.

Such innovations will allow organizations to move from static, perception-based assessments to dynamic, multi-level governance evaluation systems, enhancing both internal learning and external comparability.

### Applying Rigorous Implementation and Evaluation Designs

Most studies of shared governance rely on cross-sectional designs and perception surveys, limiting causal inference. Future research should employ rigorous designs such as longitudinal studies, stepped-wedge trials, realist evaluations, or mixed-methods implementation studies to assess how governance structures produce change over time and under varying contextual conditions. Realist approaches, in particular, are well suited to unpacking the mechanisms through which governance interacts with organizational culture, leadership, and professional roles.

Implementation strategies should incorporate structured change management processes, including stakeholder engagement, capacity building, and feedback loops. Evaluations should examine not only outcomes but also implementation fidelity, mechanisms of action, and contextual moderators.

Research should systematically explore how shared and professional governance affect workforce retention, staff well-being, job satisfaction, organizational commitment, and patient outcomes. Existing studies suggest positive associations between perceptions of governance and staff-related outcomes, including empowerment, autonomy, career motivation, and satisfaction.^12–14^,^33^ However, causal evidence remains limited, and governance variables are rarely integrated into multi-level datasets that combine workforce, patient, and organizational performance indicators. Integrating governance variables into workforce and quality datasets will help clarify their contribution to organizational performance and patient safety.

## Conclusion

Shared governance has evolved from a nursing leadership concept focused on professional autonomy and participation into a broader approach to professional and interprofessional governance in hospitals. This development reflects the increasing complexity of healthcare delivery but also reveals three structural challenges that currently limit its implementation and evaluation.

First, shared governance increasingly overlaps with related organizational frameworks, including nursing excellence recognition programs, clinical governance, quality improvement, distributed leadership, and interprofessional collaboration. Without clear boundaries, mandates, and decision rights, governance councils risk becoming parallel or symbolic structures rather than effective decision-making bodies. Second, shared governance remains strongly shaped by its nursing-centered origins, while contemporary hospitals require governance structures that meaningfully include multiple professional groups and clarify how authority is distributed across them. Third, current evaluation approaches remain too fragmented, with an ongoing emphasis on perceptions of governance culture and insufficient attention to council functioning, decision-making pathways, structural indicators, and workforce, patient, and organizational outcomes.

To remain relevant, shared governance should therefore be advanced as an explicit interprofessional decision-making system. Hospitals should define clear decision domains, align governance councils with existing organizational structures, ensure meaningful interprofessional representation, and evaluate governance beyond perceptions of culture. In practical terms, healthcare organizations can begin by mapping existing governance, quality improvement, and clinical decision-making structures, clarifying each council’s mandate and authority, and establishing feedback loops that show how council decisions influence practice and outcomes. Policymakers and system leaders can support this development by encouraging governance models that make professional participation, interprofessional authority distribution, and outcome evaluation explicit components of organizational accountability. Future research should combine measures of governance culture, council functioning, interprofessional authority distribution, decision pathways, structural characteristics, and organizational outcomes.

A more deliberate and measurable approach can help shared governance move beyond symbolic adoption toward a sustainable mechanism for strengthening professional engagement, workforce retention, interprofessional collaboration, and the quality and safety of patient care. By conceptualizing interprofessional shared governance as a decision-making system, this Expert Opinion offers a framework for connecting governance context, structures, operations, and outcomes in future implementation and evaluation.
